# Mapping urban physical distancing constraints, sub-Saharan Africa: a case study from Kenya

**DOI:** 10.2471/BLT.21.287572

**Published:** 2022-06-22

**Authors:** Heather R Chamberlain, Peter M Macharia, Andrew J Tatem

**Affiliations:** a WorldPop, Geography and Environmental Science, Building 39, University of Southampton, University Road, Southampton, SO17 1BJ, England.; b Population Health Unit, Kenya Medical Research Institute-Wellcome Trust Research Programme, Nairobi, Kenya.

## Abstract

With the onset of the coronavirus disease 2019 (COVID-19) pandemic, public health measures such as physical distancing were recommended to reduce transmission of the virus causing the disease. However, the same approach in all areas, regardless of context, may lead to measures being of limited effectiveness and having unforeseen negative consequences, such as loss of livelihoods and food insecurity. A prerequisite to planning and implementing effective, context-appropriate measures to slow community transmission is an understanding of any constraints, such as the locations where physical distancing would not be possible. Focusing on sub-Saharan Africa, we outline and discuss challenges that are faced by residents of urban informal settlements in the ongoing COVID-19 pandemic. We describe how new geospatial data sets can be integrated to provide more detailed information about local constraints on physical distancing and can inform planning of alternative ways to reduce transmission of COVID-19 between people. We include a case study for Nairobi County, Kenya, with mapped outputs which illustrate the intra-urban variation in the feasibility of physical distancing and the expected difficulty for residents of many informal settlement areas. Our examples demonstrate the potential of new geospatial data sets to provide insights and support to policy-making for public health measures, including COVID-19.

## Résumé

Avec l'apparition de la pandémie de maladie à coronavirus 2019 (COVID-19), des mesures de santé publique telles que la distanciation physique ont été mises en place afin de limiter la transmission du virus à l'origine de la maladie. Néanmoins, adopter la même approche dans toutes les régions sans tenir compte du contexte pourrait réduire l'efficacité de ces mesures et avoir des conséquences négatives imprévues, comme la perte des moyens de subsistance et l'insécurité alimentaire. Avant de planifier et de déployer des mesures utiles et adaptées à la situation en vue de ralentir la transmission au sein des communautés, il est impératif d'identifier les contraintes liées notamment aux lieux où la distanciation physique est impossible à respecter. Le présent document se concentre sur l'Afrique subsaharienne. Nous y avons présenté et évoqué les défis auxquels sont confrontés les habitants des implantations urbaines sauvages au cours de l'actuelle pandémie de COVID-19. Nous décrivons comment intégrer les nouveaux ensembles de données géospatiales pour obtenir des informations plus détaillées sur les contraintes locales liées à la distanciation physique et trouver des solutions alternatives permettant de limiter la transmission de la COVID-19 d'une personne à l'autre. Nous citons une étude de cas réalisée dans le comté de Nairobi, au Kenya, dont les résultats cartographiés illustrent les variations intra-urbaines qui déterminent la faisabilité de la distanciation physique et les difficultés que les habitants de nombreuses implantations sauvages sont susceptibles de rencontrer. Nos exemples révèlent le potentiel des nouveaux ensembles de données géospatiales dans l'analyse et l'élaboration des politiques et mesures de santé publique, y compris pour la COVID-19.

## Resumen

Con el inicio de la pandemia de la enfermedad por coronavirus de 2019 (COVID-19), se recomendaron medidas de salud pública como el distanciamiento físico para reducir la transmisión del virus causante de la enfermedad. Sin embargo, el mismo enfoque en todas las áreas, sin tener en cuenta el contexto, puede llevar a que las medidas sean de eficacia limitada y tengan consecuencias negativas imprevistas, como la pérdida de medios de vida y la inseguridad alimentaria. Un requisito previo para planificar y aplicar medidas eficaces y adecuadas al contexto para ralentizar la transmisión en la comunidad es conocer las limitaciones, como los lugares en los que no sería posible el distanciamiento físico. En este documento, centrado en el África subsahariana, se describen y discuten los desafíos a los que se enfrentan los residentes de los asentamientos urbanos informales en la actual pandemia de la COVID-19. Se describe cómo los nuevos conjuntos de datos geoespaciales pueden integrarse para proporcionar información más detallada sobre las limitaciones locales al distanciamiento físico y pueden informar la planificación de vías alternativas para reducir la transmisión de la COVID-19 entre las personas. Se incluye un estudio de caso del condado de Nairobi, Kenia, con resultados cartográficos que ilustran la variación intraurbana en la viabilidad del distanciamiento físico y la dificultad prevista para los residentes de muchas áreas de asentamientos informales. Los ejemplos que aquí se presentan demuestran el potencial de los nuevos conjuntos de datos geoespaciales para proporcionar información y apoyo a la elaboración de políticas sobre medidas de salud pública, entre ellas las relacionadas con la COVID-19.

## ملخص

مع ظهور جائحة مرض فيروس كورونا 2019 (كوفيد 19)، تمت التوصية بإجراءات للصحة العامة مثل التباعد الجسدي، وذلك للحد من انتقال الفيروس المسبب للمرض. ومع ذلك، فإن نفس الأسلوب في جميع المجالات، بغض النظر عن الوضع المحيط، قد يؤدي إلى إجراءات محدودة الفعالية ولها عواقب سلبية غير منظورة، مثل خسارة سبل العيش، وعدم الأمن الغذائي. هناك شرط أساسي لتخطيط وتنفيذ إجراءات فعالة ومناسبة للأوضاع المحيطة، لإبطاء الانتقال بين المجتمع، وهو فهم أي قيود، مثل المواقع التي لا يمكن فيها التباعد الجسدي. بالتركيز على جنوب الصحراء الكبرى بأفريقيا، نحن نحدد ونناقش التحديات التي يواجهها سكان المستوطنات العشوائية الحضرية في جائحة كوفيد 19 الجارية. نحن نصف كيفية دمج مجموعات البيانات الجغرافية المكانية الجديدة لتوفير معلومات أكثر تفصيلاً حول القيود المحلية على التباعد الجسدي، والتي يمكن أن تفيد في تخطيط الطرق البديلة لتقليل انتقال كوفيد 19 بين الأشخاص. قمنا بتضمين دراسة حالة لمقاطعة نيروبي في كينيا، مع نواتج مخططة توضح التباين داخل الحضر في جدوى التباعد الجسدي والصعوبة المتوقعة لسكان العديد من مناطق المستوطنات العشوائية. توضح الأمثلة لدينا إمكانات مجموعات البيانات الجغرافية المكانية الجديدة لتوفير رؤى ودعم لوضع سياسات لتدابير الصحة العامة، بما في ذلك كوفيد 19.

## 摘要

随着新型冠状病毒肺炎 (COVID-19) 大流行的爆发，建议采取保持社交距离等公共卫生措施，以减少导致该疾病的病毒传播。然而，在所有地区采用相同的方法，而不考虑具体情况，可能导致无法有效采取措施，并产生难以预见的负面后果，例如，丧失生计和粮食安全问题。为减缓疫情的社区传播，规划和实施因地制宜的有效措施的前提是了解所有的局限因素，例如有些场所无法保持社交距离。我们以撒哈拉以南非洲地区为重点，概述并讨论了城市非正式居住区居民在持续不断的新冠肺炎大流行中面临的挑战。我们描述了如何整合新的地理空间数据集，以进一步介绍当地在采取社交距离措施方面的局限性，并为制定替代措施规划以减少新冠肺炎在人群之间的传播提供理论信息。我们纳入了一项肯尼亚内罗毕县的案例研究，所得出的结果阐释了市内保持社交距离的可行性差异，以及许多非正式居住区的居民预期会面临的困难。我们的示例表明新地理空间数据集在为公共卫生措施（包括新冠肺炎）政策制定提供见解和支持方面具有潜力。

## Резюме

С началом пандемии коронавирусной инфекции 2019 года (COVID-19) были рекомендованы меры общественного здравоохранения, такие как физическое дистанцирование, для снижения передачи вируса, вызывающего заболевание. Однако одинаковый подход во всех областях, независимо от контекста, может привести к ограниченной эффективности мер и непредвиденным негативным последствиям, таким как утрата средств к существованию и ненадежность продовольственного обеспечения. Необходимым условием для планирования и реализации эффективных, соответствующих контексту мер по замедлению передачи инфекции внутри социума является понимание ограничений, таких как места, где физическое дистанцирование не представляется возможным. Сосредоточив внимание на странах Африки к югу от Сахары, авторы выделяют и обсуждают проблемы, с которыми сталкиваются жители городских неорганизованных поселений в условиях продолжающейся пандемии COVID-19. Авторы описывают пути интеграции новых наборов геопространственных данных, чтобы предоставлять более подробную информацию о местных ограничениях на физическое дистанцирование, которые в свою очередь могут помочь в планировании альтернативных способов снижения передачи COVID-19 между людьми. Авторы включили тематическое исследование для округа Найроби, Кения, с нанесенными на карту результатами, которые иллюстрируют внутригородские различия, связанные с возможностью реализации физического дистанцирования, и ожидаемые трудности для жителей многих районов неорганизованных поселений. Эти примеры демонстрируют потенциал новых наборов геопространственных данных для предоставления информации и поддержки разработки политики в отношении мер общественного здравоохранения, включая COVID-19.

## Introduction

The need for context-appropriate public health measures to slow or interrupt community transmission of severe acute respiratory syndrome coronavirus 2 (SARS-CoV-2) during the coronavirus disease 2019 (COVID-19) pandemic was set out in a 2021 paper.^1^ The authors described the challenges of physical distancing measures, which require keeping a distance of at least 1–2 m from other people and avoiding crowds and large gatherings. These measures were initially implemented in high-income countries, but later adopted by low- and middle-income countries without adaptation to the different contexts.^1^^,^^2^ In particular, the authors highlighted the issue of urban informal settlements in sub-Saharan Africa, drawing on examples from Kenya, Uganda and United Republic of Tanzania.^1^ While informal settlements can take different forms,^3^ urban informal settlements in these locations are often overcrowded, with amenities shared between households. In these settings, both physical and socioeconomic factors make it challenging, if not impossible, for individuals to effectively practise physical distancing.^1^^,^^4^ The feasibility of applying other interventions to reduce SARS-CoV-2 transmission also varies across settings. Conditions that can contribute to rapid community transmission of SARS-CoV-2 include insufficient ventilation of buildings; limited access to handwashing facilities or SARS-CoV-2 testing; and difficulties in practising physical distancing due to lack of space for isolation or inequalities in access to private transport.

Several studies have aimed to identify geographical variations in SARS-CoV-2 transmission risk in relation to socioeconomic and demographic factors and the physical characteristics of the built environment (the technical term is urban form). A social vulnerability index, applied at the sub-county level with national coverage, was developed for Kenya.^5^ This index incorporated indicators of socioeconomic deprivation and population characteristics, including the percentage of people living in informal settlements and in camps for internally displaced people.^5^ However, the spatial resolution at sub-county level masked heterogeneity, especially where informal settlements encompass only a small portion of a sub-county. A similar study aimed to identify high-risk locations for disease transmission in several cities (Cairo, Kinshasa and Mumbai), considering population density, building height and associated floor space area, as well as access to public toilets and water points.^6^^,^^7^ A third study in Cape Town, South Africa, identified the small spacing between dwelling units in informal settlements as posing a challenge for physical distancing.^8^ These studies have shown the intra-urban geographical variation in risk factors associated with community transmission, and highlighted informal settlements as potentially higher risk locations. The geographical coverage of these studies was limited, however. Even if it is assumed that all informal settlements in sub-Saharan African cities experience these challenges, the locations and extents of these settlements are not routinely or consistently mapped.^9^

Identifying locations within urban areas where physical distancing is not possible is therefore a key consideration in planning context-appropriate interventions to reduce the spread of SARS-CoV-2, as well as for future outbreak preparedness. We highlight how new forms of detailed geospatial data can be integrated to explore the feasibility of physical distancing within urban areas in sub-Saharan Africa, including within informal settlements.

## Population density

At high population densities, the number of potential close contacts increases. For SARS-CoV-2, which involves airborne transmission,^10^ high population densities have the potential to facilitate rapid transmission of the virus, particularly in poorly ventilated settings. If population density is defined as the number of people per geographical area, high population densities in high-income country settings are often associated with multi-storey buildings. Such buildings, while present within sub-Saharan African cities, are not the norm for residential dwellings in most cities. High population densities are more typically found in informal settlements,^11^ where dense agglomerations of one- or two-storey structures are common.

Within urban centres, high population densities can occur temporarily or can persist over longer time periods; population mobility means that the spatial distribution of the population is not constant.^12^^,^^13^ Temporary high population densities can be a singular occurrence (such as crowds associated with an event) or occurring on a regular basis (such as daily commuters at a transport hub). If not combined with guidance on adequate ventilation and correct wearing of protective masks, high population densities – whether temporary, routine or persistent – may facilitate rapid transmission. Ventilation of a space entails the introduction of clean air from outside and the removal of stale air, through natural or mechanical means. Ventilation needs are in part determined by the number of people in a space; if changes cannot be made to adequately increase ventilation, then limits on occupancy rates may be needed.^14^^,^^15^ Ventilation is particularly important in non-residential settings, such as workplaces, schools, places of worship and universities, where different households mix and crowding may occur.

To understand how population density varies geographically within urban areas, high-resolution population data sets are needed. Open-access gridded population data sets, which consist of population counts mapped to a regular grid, are available globally with population counts for grid cells of approximately 100 m x 100 m.^16^^,^^17^ These data show the variation in population density in a consistent way, both within and between cities. Such data sets enable estimates of population density to be calculated for smaller areas than publicly available population data that are linked to administrative units. National censuses are generally considered to be the standard source for population data and reflect residential population density.^18^ Alternative data sources can help capture temporal changes in population density, such as call detail records or global positioning system (GPS) data from mobile phones or location data from social media websites.^13^^,^^19^^–^^21^ Although such data sets can provide detailed information on population movements, the data are not openly available, can be representative of only a subset of the population, and may have limited geographical coverage.^22^

## Urban form

The structure and layout of a built environment also affects the feasibility of physical distancing. Quantifying measures of urban form relies on spatial data of the built environment, which until recently have generally only been available with national coverage for high-income countries. The growth in computing power, machine learning algorithms and satellite imagery have enabled the recent development of new data sets of building footprints (the outlines of buildings),^23^ covering multiple countries in Africa.^24^^,^^25^ Together with established, but increasingly detailed sources, such as OpenStreetMap,^26^ these data provide new insights into the urban form of sub-Saharan African cities. The data enable metrics such as the footprint area of buildings and the space between buildings to be estimated. However, building characteristics which may influence the feasibility of physical distancing and ventilation, such as building height, construction materials or number of rooms, are generally not available.^27^

In addition to population density and urban form, the ease of disease transmission and the feasibility of physical distancing are affected by a range of socioeconomic factors, including people’s reliance on daily wages, use of public transport and use of shared toilet facilities and water sources.^1^^,^^4^ Data on socioeconomic factors are collected through periodic household surveys, such as those conducted as part of the Demographic and Health Surveys programme.^28^ Cluster-level survey data can be interpolated to create gridded surfaces using geostatistical modelling techniques;^29^ for example the estimated percentage of the population living in households using an improved water source, mapped for grid cells of 5 km by 5 km. However, such data do not provide sufficient spatial detail to map local-level variation in socioeconomic factors at the intra-urban scale.

## Using geospatial data 

Imposing physical distancing rules to reduce community transmission of SARS-CoV-2 assumes that physical distancing is feasible everywhere. As is increasingly evident, particularly within urban areas in sub-Saharan African countries, physical distancing may not be feasible.^1^^,^^30^^,^^31^ For interventions such as physical distancing to be effective, context-specific factors need to be considered.^2^ The growing number of geospatial data sets provides increasing spatial coverage and unprecedented levels of detail, enabling greater insights at the intra-urban scale. The increasing use of online interactive maps and data portals also enable a wider audience, including policy-makers and the public, to interact directly with mapped data.^32^^,^^33^ In the context of the COVID-19 pandemic and public health management more broadly, new geospatial data sources can provide both technical specialists and decision-makers with the detailed information that is needed to plan strategies that are adapted to the specific context.

An example of harnessing recent developments in geospatial data is the ease of social (physical) distancing index developed for sub-Saharan Africa.^34^ Ease of physical distancing is calculated based on estimates of population density and the proportion of space occupied by buildings. Index values range from 0 (no expected difficulty in physical distancing) to 10 (extreme difficulty in physical distancing), mapped for small areas, which are bounded by physical features such as roads, railways, waterways. Mapping index values for small spatial units highlights intra-urban variation in the feasibility of physical distancing, with high index values often associated with informal settlements. These insights are relevant to those who are planning interventions to reduce community transmission of disease. A second example, which also uses building footprint data, calculated distances between buildings to assess the risk of disease transmission and the difficulty of practising physical distancing.^8^ This approach was limited to two informal settlements in Cape Town, but could be applied more widely given the recent development of new building footprint data sets.

**Fig. 1:**
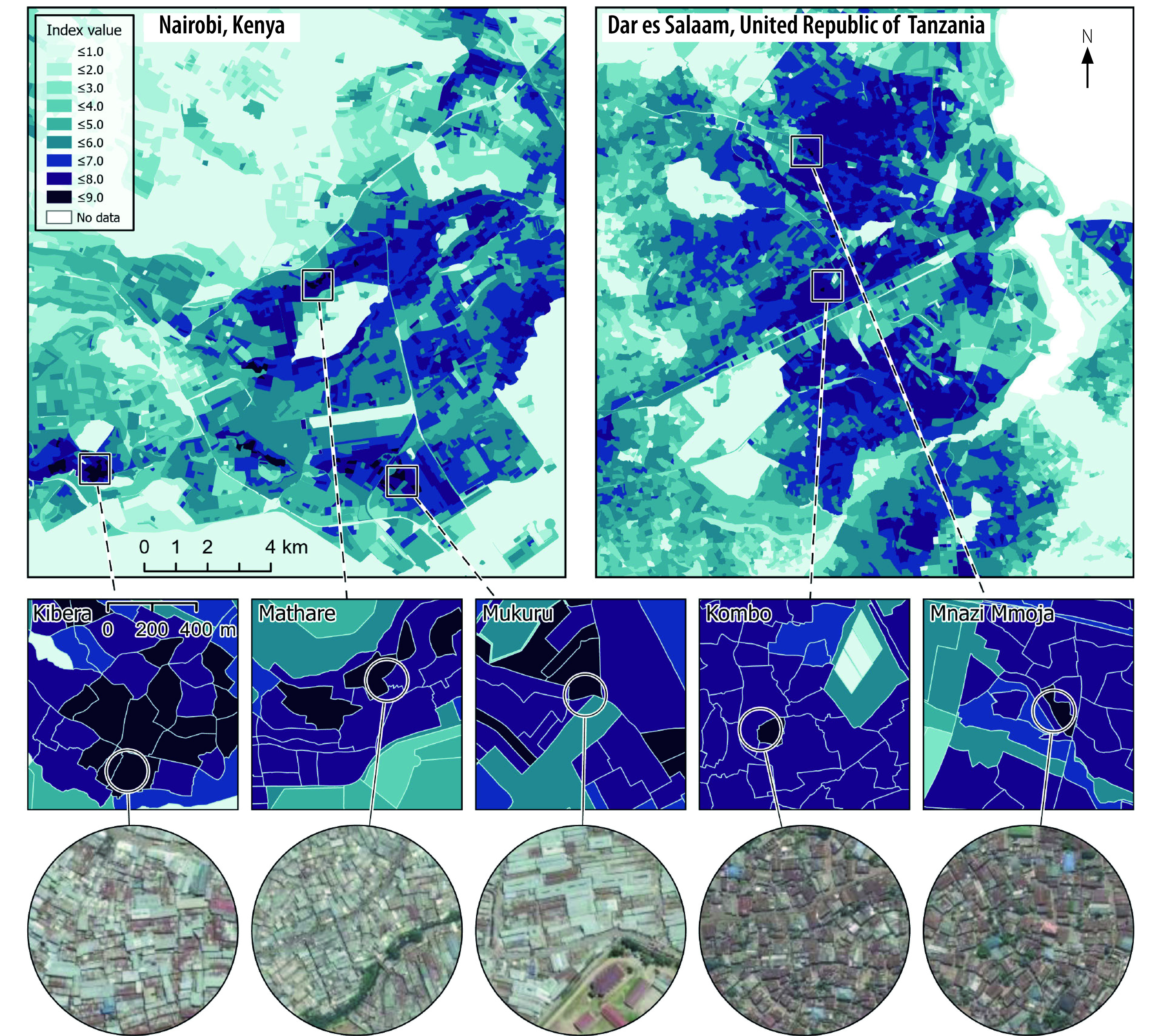
Mapped outputs for the ease of physical distancing index for Nairobi, Kenya and Dar es Salaam, United Republic of Tanzania, November 2021

Note: Large-scale overview maps (top) are shown with examples of informal settlement locations (middle) and satellite imagery (bottom) to show the urban form – the physical characteristics of the built environment – in these locations.

Integrating detailed geospatial data sets can provide new insights to support evidence from urban communities where physical distancing is impractical. Fig 1 shows mapped outputs of the ease of physical distancing index, highlighting locations where physical distancing is likely to be difficult, considering population density and urban form. In the examples shown for Nairobi, Kenya and Dar es Salaam, United Republic of Tanzania the highest index values (indicating the greatest difficulty in physical distancing) are often located within informal settlements or dense urban settlements. The examples for the informal settlements of Kibera, Mathare and Mukuru in Nairobi and the sub-wards of Kombo and Mnazi Mmoja in Dar es Salaam all have consistently high index values of 7.5–8.5. Fig. 1 also shows the geographical variation in the ease of physical distancing index over relatively small distances within the cities. The maps illustrate how uniform application of strategies such as physical distancing without consideration of the local context is likely to be ineffective in reducing community transmission of SARS-CoV-2.

Imagery source: Maxar Technologies, Westminster, United States of America.

In such settings – and other sites such as workplaces, schools, places of worship and universities – additional actions should be taken to reduce the risk of transmission of SARS-CoV-2. In particular, given the risk of airborne transmission, ventilation is important in all settings.^35^ Ensuring adequate ventilation requires estimation of the number of people in a space and, if ventilation cannot be sufficiently increased, consideration of ways to reduce room occupancy.^14^^,^^15^ In addition to ventilation and physical distancing, mask-wearing is important, particularly in settings where ventilation has been assessed to be insufficient.^36^ We now build on these examples with a more in-depth case study for Nairobi County, Kenya.

## Case study

Nairobi County is located in south-central Kenya and, despite being entirely urban, is highly diverse. There are wealthy neighbourhoods, yet the county also has 80% (814 848) of Kenya’s total urban informal settlement population of 1 016 913, over 60% (516 349) of whom live in four sub-counties (Embakasi, Kasarani, Kibra and Mathare) which are home to the major settlements of Kibera, Mathare and Mukuru (Fig. 1) and Korogocho.^37^ These informal settlements are overcrowded, with poor housing and inadequate water, sanitation and health facilities, and have historically been marginalized in government health and economic policies.^38^

After the onset of the COVID-19 outbreak in Kenya, the government introduced multiple measures to try to contain disease transmission. These measures, many of which were focused on Nairobi, aimed to reduce overcrowding and maximize ventilation, with limits on numbers or closures of venues including places of worship, schools, workplaces, sporting events, bars and restaurants,^39^^,^^40^ alongside reductions in passenger capacity on public transport.^41^ Other restrictions included partial lockdowns, curfews and limitations on movement and travel restrictions.^39^ The controls restricted population movements into and out of Nairobi,^32^ across borders with neighbouring countries^41^ and at times within some informal settlements associated with the highest reported counts of COVID-19 cases.^42^

In the informal settlements of Nairobi, it has been shown that COVID-19 control measures, especially physical distancing, may have led to food and economic insecurity for the majority of residents^42^ due to loss of income and increase in food prices. Some people were forced to skip meals^42^^,^^43^ and were unable to pay rent or service their loans. Additionally, although control measures reduced contact between people, for residents of informal settlements, the number of contacts increased with decreasing wealth.^42^ Movement and close contact is inevitable since residents have to move frequently to access services such as water, toilets and groceries, and to earn an income.^41^ Many residents in informal settlements rely on the informal sector for income and must often commute to other areas to work. Indeed, only a small proportion of residents reported that they were able to stay at home during lockdowns.^41^

**Fig. 2:**
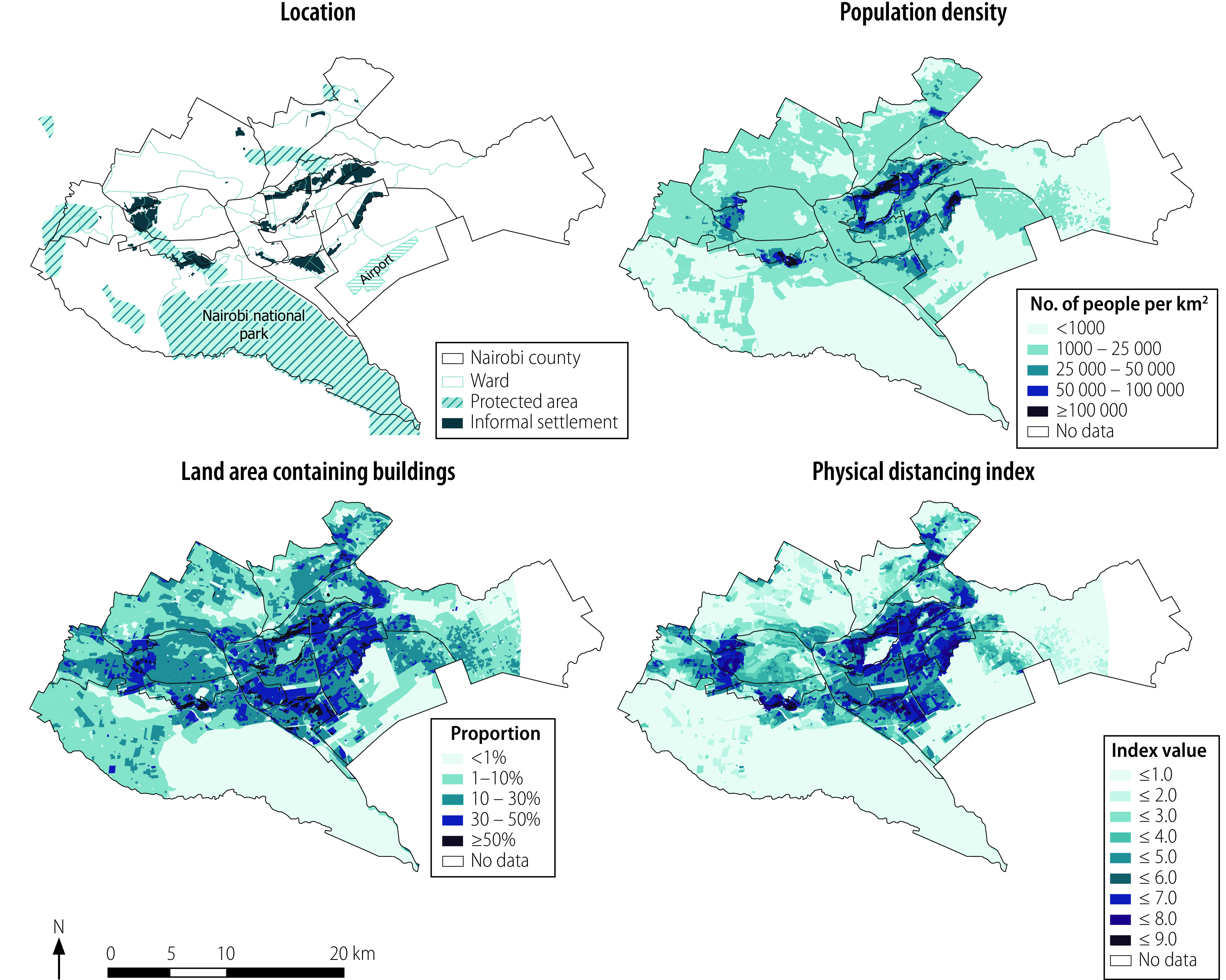
High-resolution geospatial data sets that can be used to inform context-specific COVID-19 control measures, Nairobi County, Kenya, May 2022

An approach informed by detailed geospatial layers, such as those shown in Fig. 2, could have been beneficial for improved planning and response to COVID-19 in Nairobi. The layers identify areas with low feasibility for physical distancing, broadly corresponding to the crowded urban informal settlements in Nairobi. Maintaining physical distancing and self-isolation would be almost impossible over extended periods in these areas. The maps show factors influencing the feasibility of physical distancing: population density and the proportion of land that is built-up (Fig. 2). These estimates are combined into a single index value, indicating the ease of physical distancing (Fig. 2). Darker shades in the maps represent higher values, indicating greater expected difficulty in physical distancing. These data can help identify what approaches may be appropriate and where they should be implemented.

COVID-19: coronavirus disease 2019.

Notes: The geospatial data sets are available online.^34^ Contextual features^44^ are included in the map of the location of Nairobi County and sub-county boundaries are overlaid. The ease of physical distancing index is calculated from estimates of population density and urban form, mapped for small spatial units (typically bounded by roads, rivers or other features). Darker shades in the maps represent higher values, indicating greater expected difficulty in physical distancing.

Moreover, rapid identification of such areas in Nairobi, and elsewhere, can help planners and policy-makers ensure that relevant support for residents is available. Such support could include provision of relief packages (food and cash vouchers), additional handwashing facilities,^45^ disinfection of common places such as markets^1^ and information and education on the need for ventilation. This support can help compensate people for loss of income, reduce their need to travel and reduce the risk of disease transmission where people congregate.

## Supporting policy-making

Geospatial data sets can help provide information that is needed by policy-makers and those planning public health measures. In the context of the COVID-19 pandemic, we illustrate how geographical variation in population and urban form combine to create conditions that may make physical distancing difficult. We have included examples for locations within two major cities in east Africa, focusing particularly on Nairobi. However, the mapped outputs of the ease of physical distancing index are available for 50 countries in sub-Saharan Africa, providing detailed data for most urban areas. The index data sets^34^ are publicly available. The data, with detailed metadata and documentation, can be downloaded from the WorldPop Open Population Repository^46^ and explored interactively on the GRID3 data hub.^47^ Such data sets provide novel insights that can facilitate context-appropriate measures, while minimizing the negative impact on communities and individuals. Despite the benefits, the limitations of such data sets and their utility in policy and practice need to be acknowledged.

Specific limitations of the ease of physical distancing index arise from the input data sets. Urban areas can experience rapid changes and growth,^48^ which may not be reflected in the building footprint data sets. To account for vertical urban growth would require data on building height, which is not widely available. The population density is representative of residential populations, given its basis in census enumeration.^18^ Changes in population density due to population mobility are therefore not reflected in the index values. Additional data sources, such as mobile phone records, can capture some population movements;^13^^,^^20^^,^^21^^,^^49^ however these sources are limited in their consistency and spatial or temporal availability.^22^ The index values are based on estimates of population density and urban form, but do not include other factors that will contribute to a community or individual’s experience in a location. These data sets can therefore support planners and decision-makers, and have a role in community advocacy, but should be supplemented by evidence and knowledge from other relevant sources.

Overall, these data support the perspectives of other researchers^1^ and highlight the potential for novel geospatial data sets to guide more location-specific interventions. The integration of increasingly detailed geospatial data sets can provide information at the local level on factors relevant to reduce community transmission of SARS-CoV-2. Additionally, the data sets can be used as inputs for epidemiological modelling and optimizing vaccine distribution. Longer-term, the data can aid the development of programmes to improve the health of populations in urban areas, including future pandemic preparedness and identification of locations susceptible to other risks associated with overcrowding.
